# Comparison of physiological markers, behavior monitoring, and clinical illness scoring as indicators of an inflammatory response in beef cattle

**DOI:** 10.1371/journal.pone.0302172

**Published:** 2024-04-25

**Authors:** Aiden E. Juge, Reinaldo F. Cooke, Guadalupe Ceja, Morgan Matt, Courtney L. Daigle

**Affiliations:** Department of Animal Science, Texas A&M University, College Station, Texas, United States of America; Michigan State University, UNITED STATES

## Abstract

Clinical illness (CI) scoring using visual observation is the most widely applied method of detecting respiratory disease in cattle but has limited effectiveness in practice. In contrast, body-mounted sensor technology effectively facilitates disease detection. To evaluate whether a combination of movement behavior and CI scoring is effective for disease detection, cattle were vaccinated to induce a temporary inflammatory immune response. Cattle were evaluated before and after vaccination to identify the CI variables that are most indicative of sick cattle. Respiratory rate (H_2_ = 43.08, P < 0.0001), nasal discharge (H_2_ = 8.35, P = 0.015), and ocular discharge (H_2_ = 16.38, P = 0.0003) increased after vaccination, and rumen fill decreased (H_2_ = 20.10, P < 0.0001). Locomotor activity was measured via leg-mounted sensors for the four days preceding and seven days following vaccination. A statistical model that included temperature, steps, lying time, respiratory rate, rumen fill, head position, and excess saliva was developed to distinguish between scores from before and after vaccination with a sensitivity of 0.898 and specificity of 0.915. Several clinical illness signs were difficult to measure in practice. Binoculars were required for scoring respiratory rate and eye-related metrics, and cattle had to be fitted with colored collars for individual identification. Scoring each animal took up to three minutes in a small research pen; therefore, technologies that can automate both behavior monitoring and identification of clinical illness signs are key to improving capacity for BRD detection and treatment.

## Introduction

Efforts to develop a system that can objectively identify sick cattle using non-invasive clinical signs have been ongoing for more than four decades [1]. Despite technological advances (e.g., body-mounted sensor technology and individual intake monitoring) the industry standard for detecting bovine respiratory disease (BRD) in feedlots is visual observation by pen riders, typically from horseback [2]. Several visual scoring systems have been proposed for identification of sick cattle based on clinical signs to reduce subjectivity; however, a standardized scoring system that is suitable for BRD detection in a production setting has yet to be described and validated (Table 1). A clinical illness (CI) score developed by [1] including 11 clinical signs, body temperature, and five blood parameters found differences in aggregate disease score between control cattle and those inoculated with respiratory disease-related viruses. However, the accuracy of that scoring system varied depending on which virus was used for inoculation [1], and the application of this scoring system is impractical on a large scale due to the use of blood tests. The pathogenesis of BRD in beef cattle and clinical signs of BRD were eloquently described by [3]; however, the description did not include a formalized scoring system for field use.

**Table 1:** CI score components.

	Cattle Type	Depression/Lethargy	Nasal discharge	Ocular Discharge	Fast/ Labored Breathing	Formal Decision Criteria	Cough	Temperature	Ear Droop/ Low Head	Slow Movement/Listless	Lung Auscultation	Rumen Fill	Lack of Appetite	Social Isolation	Excess Saliva	Inflamed Lymph Nodes	Conjunctivitis	Heart Rate	Abnormal Hematology	Dirty Nostrils	Diarrhea
Thomas 1977 [1]	Beef	*	*	*	*	*	*	*					*			*	*		*		*
Gardner 1999 [4]	Beef	*	*	*			*			*			*								
Buhman 2000 [5]	Beef	*	*	*		*					*	*									
Thompson 2006 [6]	Beef	*		*	*			*	*	*		*			*					*	
Schneider 2009 [7]	Beef	*	*	*	*		*	*	*				*								
Hanzlicek 2010 [8]	Beef	*			*	*		*		*								*			
Leach 2013 [9]	Beef	*	*		*									*							
Tennant 2014 [10]	Beef	*						*		*											
Toaff-Rosenstein et al 2016 [11]	Beef	*	*	*	*	*	*	*			*	*	*		*	*	*				
Pillen 2016 [12]	Beef	*	*	*	*	*	*		*	*		*		*							
Martin 2022 [13]	Beef	*	*	*	*	*		*			*								*		
McGuirk 2008 [14]	Dairy		*	*		*	*		*												
Amrine 2013 [15]	Dairy	*			*	*	*														
Love 2014 [16]	Dairy		*	*	*	*	*		*												
Buczinski et al 2014 [17]	Dairy		*	*		*	*	*	*		*										
Gaeta 2018 [18]	Dairy	*	*		*	*	*	*			*							*			
**Total**	**16**	**13**	**12**	**11**	**11**	**11**	**10**	**9**	**6**	**5**	**5**	**4**	**4**	**2**	**2**	**2**	**2**	**2**	**2**	**1**	**1**
Beef	11	11	8	8	8	6	5	7	3	5	3	4	4	2	2	2	2	1	1	1	1
Dairy	5	2	4	3	3	5	5	2	3	0	2	0	0	0	0	0	0	1	0	0	0

Criteria used for the identification of cattle afflicted with Bovine Respiratory Disease across multiple studies.

Two clinical illness scales, the Wisconsin Score and the California Score, have been developed and validated for diagnosis of BRD in dairy calves. The Wisconsin Score evaluates nasal discharge, ocular discharge, cough, ear and head position, and rectal temperature [14]. The California Score, which is a modified version of the Wisconsin Score, includes ocular discharge, nasal discharge, ear and head position, cough, breathing, and temperature [16]. Another modification of the California score includes rectal temperature and lung auscultation and excludes the evaluation of fast or labored breathing [17]. The Wisconsin Score includes body temperature and the need to induce a cough via pressure on the trachea and larynx [19]. The inclusion of these metrics requires direct contact animal handling, thus making them impractical to implement outside of dairy operations.

Clinical signs associated with BRD in dairy calves (e.g., elevated respiratory rate, fever, nasal discharge, and abnormal findings on auscultation) were more frequent in sick calves than healthy ones [18]. However, the validity of these metrics for detecting BRD in beef cattle has not been determined. Beyond the sickness response itself, the animal’s reaction to human contact could differ due to the differences between dairy and beef production systems. These differences in early life experiences and the age at which assessments are conducted can influence the efficacy of a scoring system. Beef cattle are at the highest risk for BRD at feedlot entry around six to twelve months of age [20], while dairy calves affected by BRD tend to be younger [21]. Beef cattle are also typically less habituated to humans and more exposed to predators compared to dairy cattle, and therefore could be more likely to be stressed by human presence and more skilled at masking signs of illness to avoid predation.

Even though several studies provided a specific list of clinical signs, decision criteria regarding how to evaluate these signs were often not described in detail. In studies including behavioral depression or lethargy, the most-frequently referenced clinical sign for beef cattle, only one provided an operational definition of the criterion [12]. A formal scoring system with predetermined decision points for selecting cattle to receive BRD treatment was described by [5]; however, this system included both auscultation and recording internal body temperature, thus necessitating animal handling rather than being able to conduct pen-side observation alone. A CI scoring system suitable for routine use in beef cattle needs to be derived from metrics that can be collected without handling and ideally from a distance, to minimize the effects of a perceived threat from observers [22].

Several studies in beef cattle have examined the efficacy of clinical illness scoring for identifying cases of BRD in feedlots; however, the scoring methodologies that were used varied and all scoring systems had low efficacy. A meta-analysis of seven studies estimated the overall sensitivity of CI scoring systems at 0.27 and the specificity at 0.92 [23]. A previous analysis of two of those studies [5,6] estimated sensitivity for CI scoring systems to be 0.62 and the specificity to be 0.63 [24]. The studies that were evaluated in those reviews identified cattle for BRD treatment using clinical signs including depression or lethargy, slow movement or listlessness, drooping ears, nasal discharge, ocular discharge, fast or labored breathing, lack of appetite, rumen fill, isolation from other animals, excess saliva, and dirty nostrils, as well as auscultation and temperature [4–7,9,10]. One study did not describe what signs were used to diagnose sick cattle [25]. Two more studies did evaluate clinical illness scoring systems in beef cattle and included most of the signs that were evaluated in the previous seven scoring systems listed [11,12]. An observational scoring system for BRD was proposed by [26] and was adapted by [12] to include gaunt appearance, nasal and ocular discharge, labored breathing, cough, and lack of responsiveness to humans, with a separate scale for additional signs of behavioral depression such as head carriage, slow movement, and incoordination. However, this scale has not yet been validated for accuracy in comparison with lung lesions or other confirmation of pneumonia.

### Vaccine challenge model

The initial inflammatory response to infection or injury is mediated by the innate immune system and is not pathogen-specific [27]. Pro-inflammatory cytokines, including TNF-*a* and interleukins, are released by the damaged or inflamed tissue, provoking the synthesis and release of acute-phase proteins, such as haptoglobin and serum amyloid A, from the liver [27]. A variety of physiological markers related to the innate immune response have been evaluated for prediction and detection of BRD infection. These have included white blood cell counts [28–30] interleukins and cytokines [31] and markers of oxidative stress [32]. However, across multiple studies, elevated haptoglobin levels have been found to be consistently indicative or predictive of BRD infection [29,30,32–34]. There is moderate evidence for the involvement of cortisol, however, the presence of other stressors in cattle entering a feedlot may limit the effectiveness of cortisol as a BRD biomarker [34,35].

The inflammatory immune response produced by cattle with BRD can be simulated using a vaccine against respiratory pathogens [36,37]. Vaccines can be administered via an intramuscular or subcutaneous injection, requiring the animal to undergo a minimal duration of restraint [36]. Vaccination, similarly to infection, is associated with elevated levels of haptoglobin, cortisol, TNF-*a*, insulin, leptin, ceruloplasmin, and fibrinogen [36–38]. Haptoglobin is of particular interest since it is elevated to similar levels both during BRD illness and following vaccination. Cortisol spikes rapidly after vaccination and is indicative of a stress response, which may predispose cattle to BRD illness. The response profile for both markers have been established, with peak blood cortisol levels four to eight hours after vaccination and peak haptoglobin levels one to four days after vaccination [36]. Therefore, although vaccination does not induce the long-term illness state or potential pneumonia present in BRD, it provides a limited simulation of the early stages of infection.

### Activity monitoring to detect BRD

To supplement CI scoring, non-invasive body mounted monitoring systems are being developed to detect BRD in beef cattle using data collected electronically [39]. Such systems facilitate continuous monitoring of locomotor, spatial, and ingestive behavior [2]. Studies using sensors to track locomotor and ingestive behavior [12,40,41] have found that movement and feeding behavior decrease and lying increases in sick animals. However, these models had limited specificity for respiratory illness. Findings from a vaccine challenge study, in which vaccinated heifers had lower dry matter intake post-vaccine than non-vaccinated heifers [36] suggest that reduced ingestive behavior is a generalized sign of an inflammatory response.

### Objectives

While prior research has evaluated the accuracy of clinical illness scoring for detection of BRD in dairy and beef cattle, the lack of a data-backed clinical illness scale suitable for beef cattle production conditions may have contributed to the low sensitivity reported for clinical illness scoring. Physiological markers, clinical signs, and changes in activity have been examined as sickness signs in beef cattle, however, there is a lack of knowledge about how these indicators of an inflammatory response are interrelated. The objectives of this study were to identify the most useful clinical illness signs for early detection of disease in cattle and to evaluate the relationships between clinical and behavioral markers during a known inflammatory response.

## Methods

### Approval statement

All animal procedures were approved by the Texas A&M University Agricultural Animal Care and Use Committee (AUP 2022-011A), in accordance with the Guide for the Care and Use of Agricultural Animals in Research and Teaching.

### Study animals and housing

This study was conducted at Texas A&M AgriLife McGregor Research Center using crossbred steers (n = 160). Steers were tropically-adapted crossbreeds including Angus (25%-87.5%), Hereford (<50%), Brangus (<50%), Brahman (<25%), and Nellore (<25%), and had 12.5%-37.5% total *Bos indicus* content. Steers were housed in 16 similarly designed soil-surfaced dry lot pens providing an average of 52.5m^2^ of area and 70.1cm of bunk space per steer. Half of the pens (n = 8 pens) were equipped with a stationary cattle brush (FutureCow, Sanford, FL). All 16 pens provided *ad libitum* access to water. Cattle diet was 20% corn silage, 33% sorghum, 20% corn, 7% molasses, and 2% mineral premix on a dry matter basis, with 12.9% crude protein and 71.5 mcal/kg of total digestible nutrients. Amounts per pen per day were determined day-to-day by the McGregor Research Center’s staff and ranged from 68–91 kg (6.8–9.1kg/head/day) during the study period. All steers had open access to feed in their pen’s bunk until emptied from consumption. Feed was presented daily at approximately 0800 in open J-shaped feed bunks, except for handling days, when steers were fed after handling.

### Cattle sorting

During weaning (d0), 5-month-old calves (n = 160) underwent husbandry procedures including castration, branding, and dehorning. Calves were also weighed (kg), and exit velocity (m/s), calculated from the amount of time required for each animal to travel a set distance upon being released from the chute, was measured using Rodeo Eyes sensors (FarmTex, Wylie, TX). Exit velocity is a measure of temperament that corresponds with an animal’s cortisol levels during handling and is thus indicative of the animal’s response to stress [42]. One day after all calves were weaned, calves were sorted based on castration date, body weight (BW), and exit velocity (EV) measured at weaning, then randomly assigned to one of sixteen pens so that BW and EV were balanced across pens. Five to seven focal animals within each pen (n = 96) were fitted with colored collars to facilitate visual identification. These animals included the three steers that had the fastest, slowest, and most moderate EV of the animals within that pen and two to four animals randomly selected from others within the pen.

### Vaccination, temperature, and serum samples

**Fig 1:**
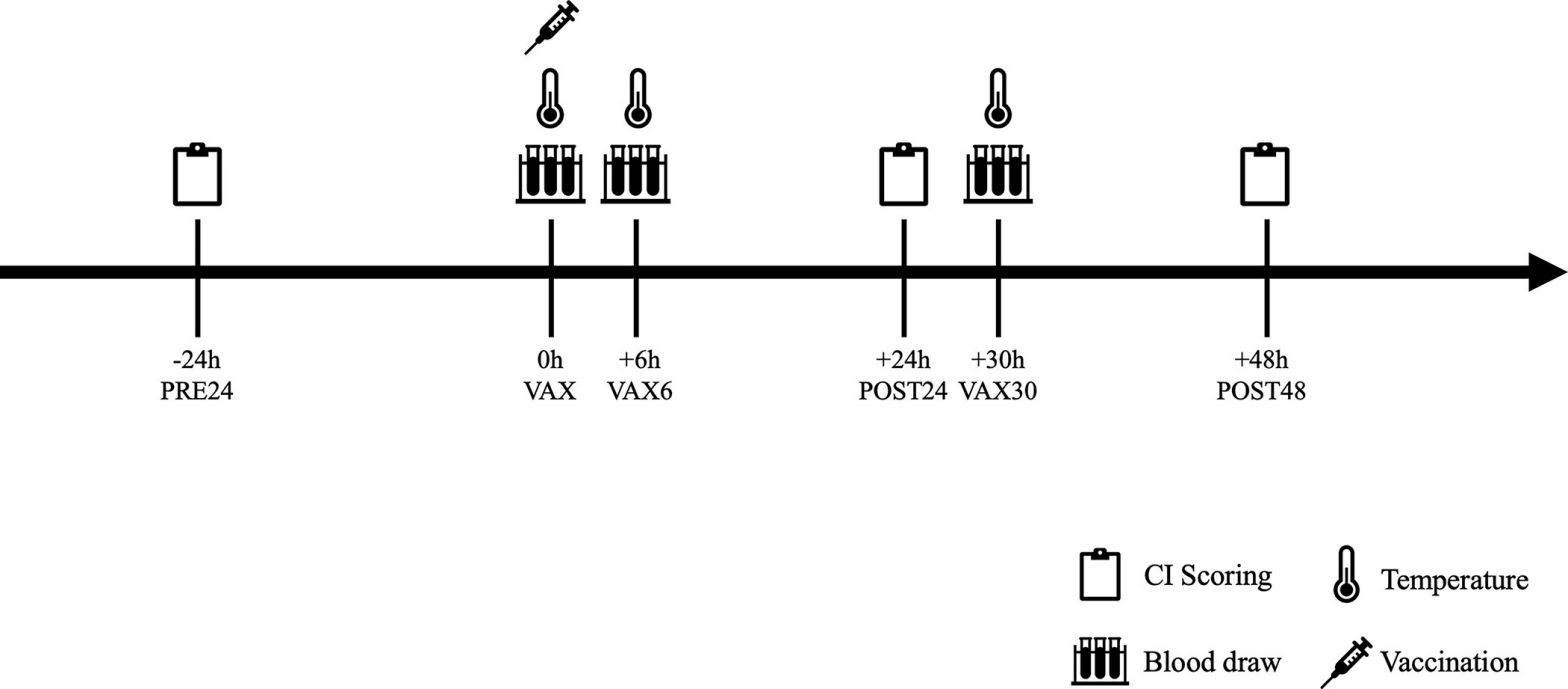
Sample collection timeline. Clinical illness scoring was conducted prior to vaccination (PRE24) and at two points after vaccination (POST24 and POST48). Rectal temperature was measured and blood samples were collected via jugular venipuncture at the time of vaccination (VAX) and at two later time points (VAX6 and VAX30).

Twenty-six days after sorting, calves were vaccinated (VAX) and serum samples were collected (Fig 1). At vaccination, calves were restrained in a hydraulic chute and each animal received 2mL of Bovi-Shield Gold One Shot (Zoetis, Parsippany, NJ) as a subcutaneous injection in the neck, using a vaccine gun with a multi-use needle. Body temperature was measured via rectal thermometer (TM99A, Cooper-Atkins, Middlefield, CT).

To validate that an immune response to vaccination had occurred, serum samples were collected from 20 focal animals. These focal animals (1–2 steers/pen) were randomly selected from the cattle that were wearing the colored collars. Blood samples were collected by jugular venipuncture using a 38mm 18g needle and two 4.0 mL red top vacutainer tubes. Study personnel wore nitrile gloves, and a fresh needle was used for each animal. Serum sampling and body temperature measurement occurred at three time points: during handling for vaccination (VAX), 5–6 hours post-vaccination (VAX6), and 29–30 hours post-vaccination (VAX30). These time points were selected to correspond with the expected peaks in cortisol and haptoglobin post handling as described by [36]. Pens of cattle were handled in the same order at each time point.

### Serum sample processing

Immediately following sample collection, tubes were inverted to mix, allowed to clot at ambient temperature, and placed on ice. Samples collected in the morning were refrigerated until processing. At the end of each day, samples were spun in a centrifuge (5804, Eppendorf, Hamburg, Germany) at 1300 rpm for 20 minutes to separate serum. Serum was aliquoted into 1.5mL microcentrifuge tubes vials using disposable pipettes and immediately frozen at -4 ˚C. After all samples were collected, samples were transported by motor vehicle 163 km to College Station, TX on dry ice. One set of samples was delivered to the Texas Veterinary Medical Diagnostic Lab for cortisol assay, one set of samples was shipped overnight on dry ice to the Kansas State Veterinary Diagnostic Lab for haptoglobin assay, and the remaining set of samples were stored at -4 ˚C. in case further analysis was required.

### Clinical illness scoring

Clinical illness scoring by live behavior observation was conducted at three time points: 24 hours before vaccination (PRE24), 24 hours after vaccination (POST24), and 48 hours after vaccination (POST48) (Fig 1). Scoring occurred immediately after cattle were fed in the morning, at the same time each day, to minimize any variability in behavior related to feeding and circadian rhythm. Clinical illness scoring included all signs described in prior studies (S1 File). If details of scoring for a sign were previously published or a published scale was available, that scale was used, with modifications if necessary. For instance, if a published scoring tool used one scale to measure multiple signs, then scales were created for each sign. If no published scale or operational definition was available, a definition was created. Clinical illness scoring was performed by 3 or 4 people, at each time point. Each observer was assigned to the same four pens at all three time points, however, at the first time point, four pens had no assigned observer, and were divided evenly among the other three observers. Prior to data collection, scorers were trained in the use of the scoring scales and datasheet. During scoring, signs were initially recorded from outside the pen, with binoculars if needed. After each pen had been scored from the outside, the scorers then entered the pen to collect the depression/lethargy score, which required evaluating the animal’s response to a human approach, and to observe any signs not visible from outside the pen. Observing eye- and nose-related signs in distant animals and observing cattle standing behind penmates required entering the pen. After scoring for each pen was completed, the observer moved on to the next pen. Coughing was recorded on a per-pen basis during the first 15 minutes of evaluation for each pen. Observers typically needed 15–25 minutes to evaluate the 5–7 focal animals in each pen.

### Locomotor behavior monitoring

At least five days prior to vaccination, 79 of the calves with collars were fitted with locomotor activity sensors (IceQube Peacock Technology, Stirling, UK), attached to a rear leg at the cannon bone via a velcro strap. Sensors were placed on five animals in all pens but one, which had four sensors. Sensors were assigned to the three animals that had the fastest, slowest, and most moderate EV within each pen and to one to two animals randomly selected from others within the pen. Data from sensors, accessed via the CowAlert database, included number of steps, standing time, lying time, and number of transitions from standing to lying and lying to standing, in 15-minute increments. The four days preceding vaccination and seven days following vaccination were included in this analysis, excluding time periods when cattle were undergoing handling procedures.

### Statistical analysis

To validate that cattle had an inflammatory immune response following vaccination, changes in cortisol and haptoglobin concentrations in serum samples were evaluated in SAS Studio (SAS Institute Inc, Cary, NC) using PROC MIXED with time as a fixed effect and animal and pen as random effects. Means were compared via Least Squared Means with a Bonferroni correction. Differences were considered statistically significant at P < 0.05 for all tests.

Cattle body temperature was analyzed likewise.

Locomotor data for each animal was averaged daily. To exclude data from times when cattle were being handled, and to control for circadian behavior patterns, locomotor activity over 11 days, from 4 days prior to vaccination through 7 days after vaccination, was analyzed during the 14h period from 1600h to 0600h on days when no handling activity occurred. Days were numbered relative to vaccination (e.g, PRE-0 refers to the time period from 1600h the day before vaccination to 0600h the morning of vaccination, while POST+0 refers to the time period from 1600h the day of vaccination to 0600h the morning after vaccination). A General Mixed Model (PROC MIXED) was used to identify changes across time in steps, standing time, and transitions from standing to lying. Time was included as a fixed effect with animal and pen as random effects. Means were compared via Least Squared Means with a Bonferroni correction. Due to some pedometers becoming detached during the study period, data from 76 or 77 animals was available at each time point.

Analyses of clinical illness score items utilized a nonparametric approach due to lack of normality. PROC NPAR1WAY in SAS was used to perform a Kruskal-Wallis test for changes in each variable (E.g., dirty nostrils, saliva, conjunctivitis, labored breathing, ear droop, lowered head, isolation from other cattle, behavioral depression, nasal discharge, ocular discharge, respiratory rate, rumen fill, and posture) among scoring times. Means were compared via the DSCF method. Kruskal-Wallis tests were also used to identify pen and observer effects. Rumen fill was reverse scored, in that a lower score was expected to indicate an increased extent of illness, while higher scores on all other signs were expected to indicate illness.

Spearman correlation coefficients (PROC CORR) between CI score variables evaluated at PRE24, POST24, and POST48 were calculated to evaluate the relationship between CI signs. Since many illness signs were correlated, a discriminant analysis was used to identify sickness signs that were most useful for distinguishing between pre-vaccination time point (healthy model) and post-vaccination time point (sickness model) animals. However, prior to vaccination, several animals already had a body temperature above the normal range of 36.7–39.1 ⁰C (Robertshaw, 2004). The presence of potentially sick cattle in the pre-vaccination measurements presents a problem in evaluating signs reflective of the vaccine-induced sickness response. Therefore, data from nine animals that had a body temperature greater than or equal to 40 ⁰C at VAX were excluded from the discriminant modelling process.

Time relative to vaccination was the grouping variable for the discriminant model. Pre-vaccination measurements included data from the PRE24 CI scoring, temperature at VAX, and locomotor data for the 45h preceding PRE24. Post-vaccination measurements included data from POST48 CI scoring, temperature at VAX30, and locomotor data for the 45h following POST48. A second model was created using only data from PRE24 and POST48 CI scoring. PROC STEPDISC was used descriptively to select variables in a stepwise fashion. PROC DISCRIM was used with a nonparametric k-nearest neighbors method to construct a discriminant model. Cross-validation was performed to assess model performance, and false positive rate (FPR), false negative rate (FNR), sensitivity (SE) and specificity (SP) were calculated.

## Results

### Temperature

Cattle body temperature differed among time points (F_2, 190_ = 186.30, P < 0.0001). Temperature at VAX (39.39 ± 0.05 ⁰C) was lower than temperature at VAX6 (40.51 ± 0.05 ⁰C, P < 0.0001) and temperature at VAX30 (40.28 ± 0.05 ⁰C, P < 0.0001). Body temperature at VAX30 was less than temperature at VAX6 (P = 0.0002).

Ambient temperature ranged from 22.7–27.2 ⁰C at VAX, 28.3–30.6 ⁰C at VAX6, and 33.3–34.4 ⁰C at VAX30. Weather conditions were sunny at VAX and VAX30, and cloudy at VAX6, with no precipitation.

### Serum cortisol and haptoglobin

Twenty serum samples from each time point were tested for cortisol and haptoglobin levels. As expected, mean haptoglobin differed among time points (F_2, 33_ = 209.14, P < 0.0001). Haptoglobin was greater at VAX30 (95.8 ± 3.23 mg/dl) than at VAX (17.30 ± 2.80 mg/dl, P < 0.0001) and VAX6 (19.30 ± 2.80 mg/dl, P < 0.0001). Mean cortisol concentrations also differed among time points (F_2, 38_ = 9.85, P = 0.0004) where serum cortisol was higher at VAX30 (4.26 ± 0.30 ug/dl) than at VAX (3.01 ± 0.30 ug/dl; P = 0.0002).

### Clinical illness signs

No differences were observed among time points for dirty nostrils (H_2_ = 3.54, P = 0.170), saliva (H_2_ = 0.52, P = 0.771), conjunctivitis (H_2_ = 5.23, P = 0.073), labored breathing (H_2_ = 3.54, P = 0.171), ear droop (H_2_ = 1.99, P = 0.370), lowered head (H_2_ = 1.99, P = 0.369), isolation from other cattle (H_2_ = 0.69, P = 0.710), or behavioral depression (H_2_ = 2.00, P = 0.368).

Nasal discharge (H_2_ = 8.35, P = 0.015) scores differed across time points. Nasal discharge scores were lower at PRE24 (N = 96, M = 0.188) than at POST48 (N = 96, M = 0.406, P = 0.015), but neither differed from scores at POST24 (N = 94, M = 0.330, P = 0.658). Ocular discharge scores changed over time (H_2_ = 16.38, P = 0.0003). Ocular discharge scores were lower at PRE24 (N = 96, M = 0.063) than at POST24 (N = 94, M = 0.426, P = 0.0001) and at POST48 (N = 96, M = 0.292, P = 0.011).

Respiratory rate, as measured by the number of breaths per 10s (H_2_ = 43.08, P < 0.0001) differed over time. Respiratory rate was lower at PRE24 (N = 77, M = 5.883) than at POST24 (N = 89, M = 7.315, P < 0.0001) and at POST48 (N = 80, M = 7.900, P < 0.0001). Rumen fill scores (H_2_ = 20.10, P < 0.0001) were higher at PRE24 (N = 96, M = 4.021) than POST24 (N = 90, M = 3.533, P < 0.0001) and POST48 (N = 96, M = 3.651, P = 0.002).

Animal posture (e.g., lying down) differed among time points (H_2_ = 18.52, P < 0.0001). More animals were lying down at POST24 (N = 96, M = 0.094) than PRE24 (N = 96, M = 0.00, P = 0.006) or POST48 (N = 96, M = 0.00, P = 0.006).

Coughs were evaluated on a per-pen basis, and number of coughs varied across time points (H_2_ = 20.46, P < 0.0001). More coughs were observed at POST48 (N = 16, M = 10.19) than PRE24 (N = 16, M = 1.88, P = 0.0003) or POST24 (N = 16, M = 3.25, P = 0.001).

### Locomotor behavior over time

**Fig 2:**
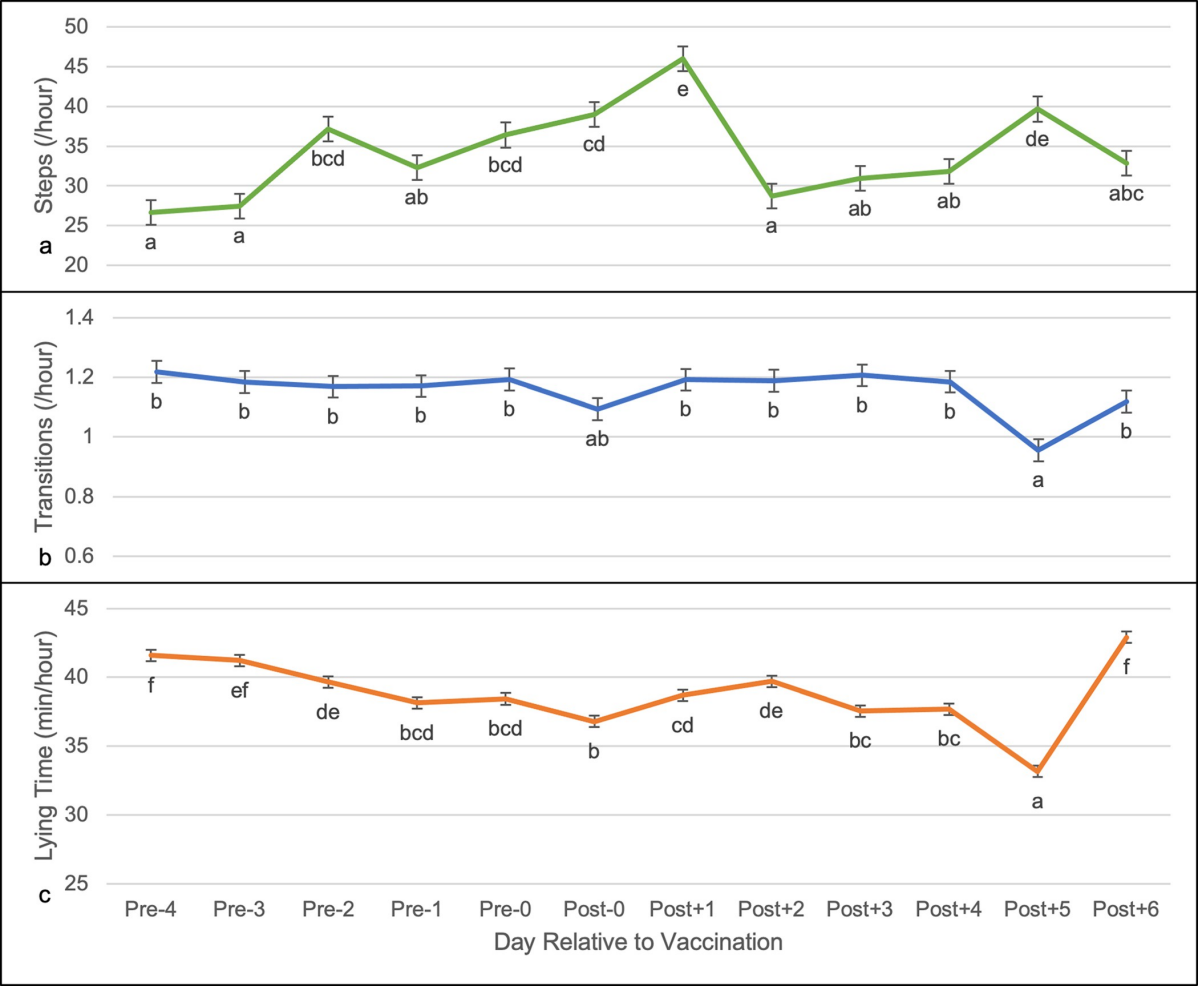
Daily locomotor activity. Number (average ± SEM) of a) steps (count/h), b) transitions (count/h) between standing and lying, and c) time spent lying (min/h) in cattle before and after vaccination during the 14h period each day when cattle were not handled. Letters indicate significant differences at **α** = 0.05.

The mean number of steps per hour differed among days (F_11, 840_ = 17.43, P < 0.0001). Cattle took the most steps on Post+1 and Post+5, and the least steps on Pre-4, Pre-3, and Post+2 (Fig 2A). Number of transitions between lying and standing per hour also varied over time (F_11, 840_ = 5.57, P < 0.0001). There were fewer transitions on Post+5 than on any other day except Post+0 (Fig 2B). Lying time, measured in mean minutes per hour (F_11, 840_ = 48.81, P < 0.0001) was least on Post+5 and greatest on Post+6, Pre-4, and Pre-3 (Fig 2C).

### Relationships among clinical illness signs

Spearman rank correlations evaluated relationships between clinical illness variables (Table 2). Time relative to vaccination was positively associated with increased nasal discharge, ocular discharge, respiratory rate, and decreased rumen fill. Nasal discharge was positively correlated with ocular discharge, dirty nostrils, conjunctivitis, saliva, respiratory rate, and depression; and was negatively correlated with rumen fill. Ocular discharge was positively associated with conjunctivitis, respiratory rate, labored breathing, ear droop, lowered head, and decreased rumen fill. Dirty nostrils were positively correlated with ear droop. Saliva was positively correlated with labored breath and depression. Increased respiratory rate was associated with higher conjunctivitis scores, higher labored breathing scores, and presence of a lowered head. Labored breathing was positively correlated with ear droop, lowered head, isolation, depression, and lying posture, and negatively correlated with rumen fill. Lowered head was associated with the presence of ear droop, isolation, depression, and decreased rumen fill. Isolation was positively correlated with lying posture.

**Table 2:** Correlations among clinical illness signs.

Variables	Nasal Discharge	Ocular Discharge	Dirty Nostrils	Conjunctivitis	Saliva	Respiratory Rate	Labored Breath	Ear Droop	Lowered Head	Isolated	Depression	Rumen Fill
**Ocular Discharge**	**0.251**											
**Dirty Nostrils**	**0.116**	0.054										
**Conjunctivitis**	**0.218**	**0.337**	-0.039									
**Saliva**	**0.165**	0.089	-0.047	-0.026								
**Respiratory Rate**	**0.228**	**0.289**	0.066	0.198	0.001							
**Labored Breath**	0.019	**0.186**	0.40	0.000	**0.280**	**0.182**						
**Ear Droop**	-0.031	**0.143**	**0.149**	0.000	0.000	0.000	**0.497**					
**Lowered Head**	0.088	**0.172**	0.116	-0.043	0.100	**0.145**	**0.474**	**0.236**				
**Isolated**	0.083	0.031	0.104	-0.040	-0.022	0.081	**0.149**	-0.011	**0.124**			
**Depression**	**0.130**	-0.028	-0.024	0.000	**0.497**	0.109	**0.497**	-0.004	**0.236**	-0.011		
**Rumen Fill**	**-0.188**	**-0.275**	-0.101	0.021	-0.002	-0.022	**-0.143**	-0.061	**-0.133**	-0.059	-0.060	
**Posture**	-0.093	0.022	-0.072	-0.043	-0.022	-0.018	**0.149**	-0.011	0.040	**0.197**	-0.011	-0.094

Spearman-Rank correlations among clinical illness signs collected from 96 drylot-housed steers 24 hours before, 24 hours after, and 48 hours after vaccination for Bovine Respiratory Disease. Statistically significant relationships (P < 0.05) are in bold.

### Predictive value of clinical illness signs

An initial stepwise discriminant analysis identified seven variables of interest: body temperature, steps per hour, lying time per hour, rumen fill, lowered head, respiratory rate, and excess saliva. Excluding observations with missing data, the model included data from 59 animals pre-vaccination and 49 animals post-vaccination. A nonparametric cross-validation procedure indicated that the model had an estimated error rate of 0.093 (FPR = 0.085, FNR = 0.102, SE = 0.898, SP = 0.915), indicating that with a 1:1 ratio of sick to healthy cattle, the model would correctly classify 90.7% of animals.

A second analysis was performed using only clinical illness signs that could be assessed visually (e.g., excluding temperature, steps, lying time, and transitions). Stepwise variable selection identified respiratory rate, rumen fill, and excess saliva as the best indicators. This model included data from 70 animals pre-vaccination and 71 animals post-vaccination. The cross-validation error rate was 0.298 (FPR = 0.300, FNR = 0.296, SE = 0.704, SP = 0.700).

### Pen and observer effects

There were pen effects on the expression or prevalence of several clinical signs; however, observers were assigned to the same group of pens at each time point, so pen and observer effects are not independent. Pens differed in their average nasal discharge (H_15_ = 29.03, P = 0.016), ocular discharge (H_15_ = 60.77, P < 0.0001), respiratory rate (H_15_ = 33.98, P = 0.003), rumen fill (H_15_ = 70.58, P < 0.0001), and posture (H_15_ = 26.17, P = 0.036) scores.

Scores differed among observers for rumen fill (H_3_ = 82.52, P < 0.0001), dirty nostrils (H_3_ = 16.25, P = 0.001), nasal discharge (H_3_ = 7.92, P = 0.048), ocular discharge (H_3_ = 36.97, P < 0.0001), respiratory rate (H_3_ = 16.85, P = 0.0008), labored breathing (H_3_ = 9.98, P = 0.019), and lowered head (H_3_ = 8.96, P = 0.029).

## Discussion

In this study, the best indicators of sickness included body temperature, daily step count, daily lying time, rumen fill, head position, respiratory rate, and excess saliva. These results emphasize the need to integrate multiple approaches to health monitoring to accurately identify illness in cattle. Consequently, a multi-pronged approach is required to collect and interpret these types of data. Other potentially relevant illness signs included nasal discharge, ocular discharge, animal posture, and coughing. Transitions between standing and lying, dirty nostrils, conjunctivitis, and depression were not directly linked with illness state.

Serum biomarkers indicated that vaccination was effective in inducing a temporary inflammatory response in cattle, as expected. Haptoglobin increased as expected from baseline to after vaccination, as did cattle body temperature. In contrast to a previous study describing serum cortisol levels after vaccination [36] cattle exhibited an increase in cortisol from baseline to 30h after vaccination, rather than a clear peak at 4-8h after vaccination. However, serum cortisol levels can change rapidly in response to extraneous factors, including handling stress [43]. Although pens of cattle were handled in the same order at each sample collection time point, so that animals would spend a similar amount of time in holding areas, the repeated handling may have compounded the stress and subsequent cortisol levels experienced in response to vaccination.

Body temperature increased after vaccination, and subsequently decreased slightly, but remained higher than baseline. This corresponded with the expected inflammatory response resulting from vaccination. Because body temperature decreased from VAX6 to VAX30 while ambient temperature increased, it is unlikely that weather conditions were responsible for the changes in body temperature.

Prevalence of several clinical illness signs increased after vaccination. Ocular discharge, nasal discharge, and respiratory rate increased, rumen fill decreased, and more animals were observed in a lying posture after vaccination. While the presence of most other sickness-related variables increased numerically, they were nonetheless rare even after vaccination. The presence of all illness sign was positively correlated with presence of at least one other illness sign and/or negatively correlated with rumen fill. This suggests that all signs included in this study are part of multiple interrelated clusters of symptoms. Given the rarity of many of the clinical illness signs in this model, this study’s sample size was likely insufficient to identify whether those signs are effective indicators of an inflammatory response when they are present. Additionally, these results should be interpreted cautiously, since observers were not blinded to animals’ vaccination status.

Changes in locomotor behavior over time were statistically significant but difficult to interpret. Lying fluctuated across the measurement period. While there was a slight increase in lying behavior two days post-vaccination, the largest change occurred between five and six days post-vaccination, during which lying behavior dropped and subsequently increased This may have been due to temperature change, as the daily average temperature fell from 30 ˚C to 16.7 ˚C over the same period [44]. Average transitions per hour did not vary between most days but had a similar pattern of decrease five days post-vaccination and recovery six days post-vaccination. Number of steps per day had the greatest variation throughout the study period, with a notable increase one day after vaccination and subsequent decrease two days after vaccination. Previous studies monitoring behavior prior in cattle that spontaneously developed BRD found that cattle that developed BRD took fewer steps and had fewer bouts of lying than cattle that did not develop BRD [12,40]. Further, sick animals spent less time eating than healthy animals. A model constructed from activity and feeding data was able to predict development of illness nine days in advance, with a sensitivity of 0.79 and specificity of 0.81 [40]. Despite having a relatively high false positive rate of 0.47, administering antibiotic treatment only to animals that were predicted to become sick instead of utilizing whole herd metaphylaxis would substantially reduce antibiotic use [40]. Another study, in calves fitted with collars to monitor rumination and locomotor activity, found that daily locomotor activity decreased three days prior to BRD diagnosis, while rumination decreased six days prior to diagnosis [40]. However, because BRD is not caused by infection with a single pathogen, there is not a consistent incubation period between inciting events and onset of clinical signs [20]. Therefore, the timing of the beginning of the inflammatory response in the aforementioned studies is unknown; whereas in this study only a limited inflammatory response occurred, limiting the comparisons that can be drawn with regard to changes in activity level.

Coughing frequency per pen increased from baseline to post-vaccination. Coughing has previously been identified as the best indicator of respiratory disease in both beef and dairy calves [19] but evaluating this metrics on a per-animal basis in a large herd is not feasible since coughs are of short duration and may be difficult to localize to a specific animal. Body temperature increased during the inflammatory response, and is therefore a useful health indicator, but is also difficult to monitor in animals that are not handled regularly.

When a discriminant analysis was conducted, a model that incorporated temperature, CI signs, and locomotor activity achieved 90.7% accuracy in correctly classifying animals by sickness state. The model correctly classified 89.8% of post-vaccination animals and 91.5% of pre-vaccination animals. These results were obtained via a cross-validation method that involved excluding a given animal’s data from the model then classifying that animal, meaning that theoretically similar accuracy should be obtained when applied to different cattle under similar circumstances. These results compare favorably to traditional CI scoring [23], and slightly exceeds the performance of the locomotor behavior-based model developed by [40]. While promising, this should be interpreted with caution, since the model has not been tested in cattle genuinely ill with BRD.

A model using visual CI scoring criteria alone had limited effectiveness in distinguishing between pre-vaccination and vaccine-challenged cattle. The best model using only visual CI scoring criteria included respiratory rate, rumen fill, and saliva and was 70.2% accurate in classifying observations as coming from the animal from either pre- or post- vaccination. The sensitivity of this model was higher than that typical of clinical illness scoring, however, the specificity was comparable or lower. Therefore, a score using these criteria would be likely to accurately identify more cattle as sick than other clinical illness scores but would also inaccurately identify more healthy cattle as sick.

There were practical constraints involved in measuring many of the clinical illness signs, including those that were selected as key markers of illness, such as posture, rumen fill, and respiratory rate. Posture and rumen fill could be rapidly observed without the need for magnification. However, rumen fill observations require stockperson training and consistent timing relative to feed delivery, further, animal posture typically changes rapidly upon approach by humans increasing the difficulty associated with evaluating this metric. For most nasal discharge, ocular discharge, and saliva observations, binoculars were necessary and required the animal to be facing a specific direction for evaluation. While not insurmountable, these challenges provide additional constraints on visual CI scoring, and the challenges experienced may be exacerbated by evaluating from horseback.

Respiration rate was difficult and time-consuming to measure. For 42 of 288 observations (14.6%), respiration rate could not be determined, even in an experimental design with relatively small numbers of cattle and the opportunity to observe animals at close range, with magnification, for as much time as necessary. Respiration rate measurements also varied between observers, suggesting a high potential for error. This supports the findings of [45] who reported similar problems of inaccuracy and time-intensiveness when conducting visual observations of respiration rate. Therefore, even though respiration rate is informative regarding the current health status of an animal, measuring respiration rate in the field is unlikely to be a feasible criterion as part of a feedlot illness scoring tool.

Conjunctivitis did not change over time; however, conjunctivitis was positively correlated with nasal discharge, ocular discharge, and respiratory rate, all of which did increase after vaccination. Although there were no differences in conjunctivitis scores between observers, conjunctivitis was reported to be the second-most-difficult criterion to measure. Observers expressed that the white of the eye was not clearly visible in all animals and required close-range observation even with binoculars to accurately determine. While less time-consuming than measuring respiratory rate, conjunctivitis scores could not be determined for 48 observations (16.7%), accounting for more incomplete scores than any other metric. This suggests that conjunctivitis scoring would have limited feasibility for practical application even if it were found to be a good indicator of illness.

There were observer and pen differences in accuracy for many CI score items. Each observer was assigned to the same block of pens at each time point to control for observer effects when analyzing the effect of time relative to vaccination. It is therefore not possible to fully distinguish between pen effects and observer effects in this design. However, low inter-rater reliability is a known problem in clinical illness scoring. A study in veal calves using the Wisconsin Score found that even among experienced observers, only induced cough, ear droop, and head tilt had acceptable interrater reliability, while reliability for ocular discharge, nasal discharge, and abnormal respiration were low [46]. These correspond to the areas in which observers’ scores differed the most in this study, indicating that lack of inter-rater reliability was likely a factor in apparent pen differences.

Assessing which illness signs are good predictors of a sickness response also proved challenging in part because few animals displayed symptoms. Cattle are a prey species, and therefore avoid displaying outward signs of illness where possible to reduce predation risk [47]. Beef cattle’s wariness of humans further contributes to difficulty in assessing clinical illness signs [22]. Additionally, vaccination does not induce prolonged or severe illness, reducing the likelihood that signs such as labored breathing would be present in this study. Many illness signs were present in too few cattle to truly determine their efficacy as positive indicators of disease. However, presence of most clinical illness signs was linked to the presence of at least one other illness sign. This suggests that all these illness signs have the potential to occur in a sick animal, even though they may not occur in most sick animals, which corresponds with previous findings that indicate CI scoring has high specificity, but low sensitivity. Most animals that display illness signs are sick, but many animals that are sick do not display illness signs. In an applied context, it may be advisable to err on the side of treating cattle that display any signs of illness; however, this runs the risk of administering unnecessary antibiotics, contributing to increasing rates of antibiotic resistance [48].

Emerging technologies may have the potential to mitigate some of the challenges inherent to CI scoring in cattle by monitoring signs that are not feasible for human observation. This is already the case regarding the use of sensors to track locomotor activity and feeding behavior. As discussed by [2], technologies that continuously track cattle behavior can reduce the time needed to monitor cattle on an individual basis, and do not induce changes in behavior like those caused by human presence. Two studies that used accelerometers to monitor calves’ steps and lying behavior identified decreased locomotor behavior six to nine days prior to clinical signs [12,40]. However, similar behavioral changes were observed in animals that developed lameness [41], indicating that activity monitoring may be effective for identifying animals in need of veterinary care, but may not be able to distinguish between potential causes of poor health in cattle. Ear tag accelerometer devices are also able to track feeding behavior in beef cattle [49] and have been shown to be an effective method of activity monitoring for BRD detection in dairy calves [50].

In addition to behavioral indicators of sickness, other precision livestock farming technologies identify illness signs directly. Research in dairy calves has resulted in an algorithm that can detect coughs from audio recordings [51]. However, there were pronounced tradeoffs between sensitivity and specificity [51] and it is unlikely that an audio-based system would work well in beef cattle, where animals are able to commingle as a group and are typically housed outside. Remote body temperature monitoring via infrared is possible [52], although the technical and logistical considerations in capturing that information requires sensor equipment and a way to indicate which animals need treatment after identifying those that are flagged as having a high body temperature. An implantable microchip that reports temperature is also available but has limited accuracy, and RFID implants are rarely used in beef cattle due to migration risk, as well as cost [53]. The need for close-range interaction to scan each animal also limits the temperature-sensing chip’s utility. GrowSafe feed bunks, which track individual animal feeding behavior, can contribute to sickness detection [54] and could be used in lieu of visual estimates of rumen fill. Efforts have been made to develop wearable respiratory rate sensors for cattle [45,55]. However, poor ease of use and high cost per animal currently limit implementation of sickness detection technologies in beef cattle feedlots [56]. Auscultation [5], radiography [57], ultrasound [29], and pathogen-specific laboratory tests [18] are available for confirming diagnoses, but are not suitable for screening large numbers of animals. Ultimately, due to the limitations of both visual clinical illness scoring and precision livestock technology implementation in beef cattle, a combination of skilled observation and emerging technologies will be needed to effectively provide targeted BRD treatment.

## Supporting information

S1 File CI score rubric. Clinical illness scoring criteria and sources.
(XLSX)

S2 File Clinical illness score data. Clinical illness score and temperature observations.
(XLSX)

S3 File Locomotor data. Locomotor behavior data as downloaded from Iceqube pedometers.
(XLSX)
